# Towards *stimulando* time-resolved infrared spectroscopy to study intermittent light-stimulated CO_2_ hydrogenation

**DOI:** 10.1039/d5fd00150a

**Published:** 2026-01-06

**Authors:** Floor A. Brzesowsky, Mees R. Emond, Jules van Leusden, Ramon Oord, Peter de Peinder, Bert M. Weckhuysen, Matteo Monai

**Affiliations:** a Inorganic Chemistry and Catalysis Group, Institute for Sustainable and Circular Chemistry, Department of Chemistry, Faculty of Science, Utrecht University Universiteitsweg 99 3584 CG Utrecht The Netherlands m.monai@uu.nl

## Abstract

Resonant catalysis theory predicts that applying an intermittent stimulus, such as charge, strain, heat or light, at frequencies on a timescale higher than the catalytic turnover frequency, can enhance activity by orders of magnitude and improve selectivity. However, experimental evidence of resonant catalysis is unfortunately lacking. This is partly due to the fact that the effect of intermittent stimulation on catalysts and reaction intermediates is not well understood. To address this challenge, time-resolved “*stimulando*” spectroscopic methods are needed to observe catalysts under operating conditions and during stimulation. Here, we use diffuse reflectance infrared Fourier-transform spectroscopy (DRIFTS) to study the effect of intermittent light stimulation on the catalytic hydrogenation of CO_2_ over Ni–Ga-based catalyst materials as a model reaction. Previous research has shown that light can cause CO desorption and change the reactivity of formate intermediates during reaction. Since CO and formate species are believed to be active intermediates in CO_2_ hydrogenation, understanding how intermittent light affects their binding energy and surface coverage can provide insights into how to effectively stimulate catalysts to possibly achieve resonant catalysis. Ni_3_–Ga/SrTiO_3_ catalysts were synthesized using incipient wetness impregnation and tested under CO_2_ hydrogenation reaction conditions. *Stimulando* DRIFTS measurements were performed using both steady-state and rapid-scan DRIFTS to study the effect of continuous and intermittent ultraviolet (UV) light stimulation. Steady-state DRIFTS revealed that the surface coverage of CO, formate, and carbonate intermediates decreased reversibly upon UV illumination, with each species exhibiting distinct timescales to reach steady state (ranging from seconds to minutes). Furthermore, rapid-scan DRIFTS with millisecond time resolution demonstrated that spectral changes generally occurred faster when switching the UV light on compared to when switching it off in steady-state experiments. However, when using intermittent light at a 1 Hz on/off frequency, the rates of change for spectral features upon light switching on and off became comparable. This showcases the need to study catalyst stimulation under intermittent stimulation to capture the dynamic response of the system at the limit cycle. Despite the observed changes in the coverage of surface species, the CO_2_ hydrogenation performance of the catalyst was not significantly affected under the conditions studied herein. The *stimulando* spectroscopy method showcased here provides valuable insights for adjusting light stimulation parameters, such as intensity, duty cycle and light wavelength, paving the way to more effective catalyst stimulation.

## Abstract 2

This study showcases a novel *stimulando* spectroscopic approach for investigating the effect of intermittent catalyst stimulation, with the goal of providing more efficient stimulation strategies for dynamic and resonant catalysis.

## Graphical abstract

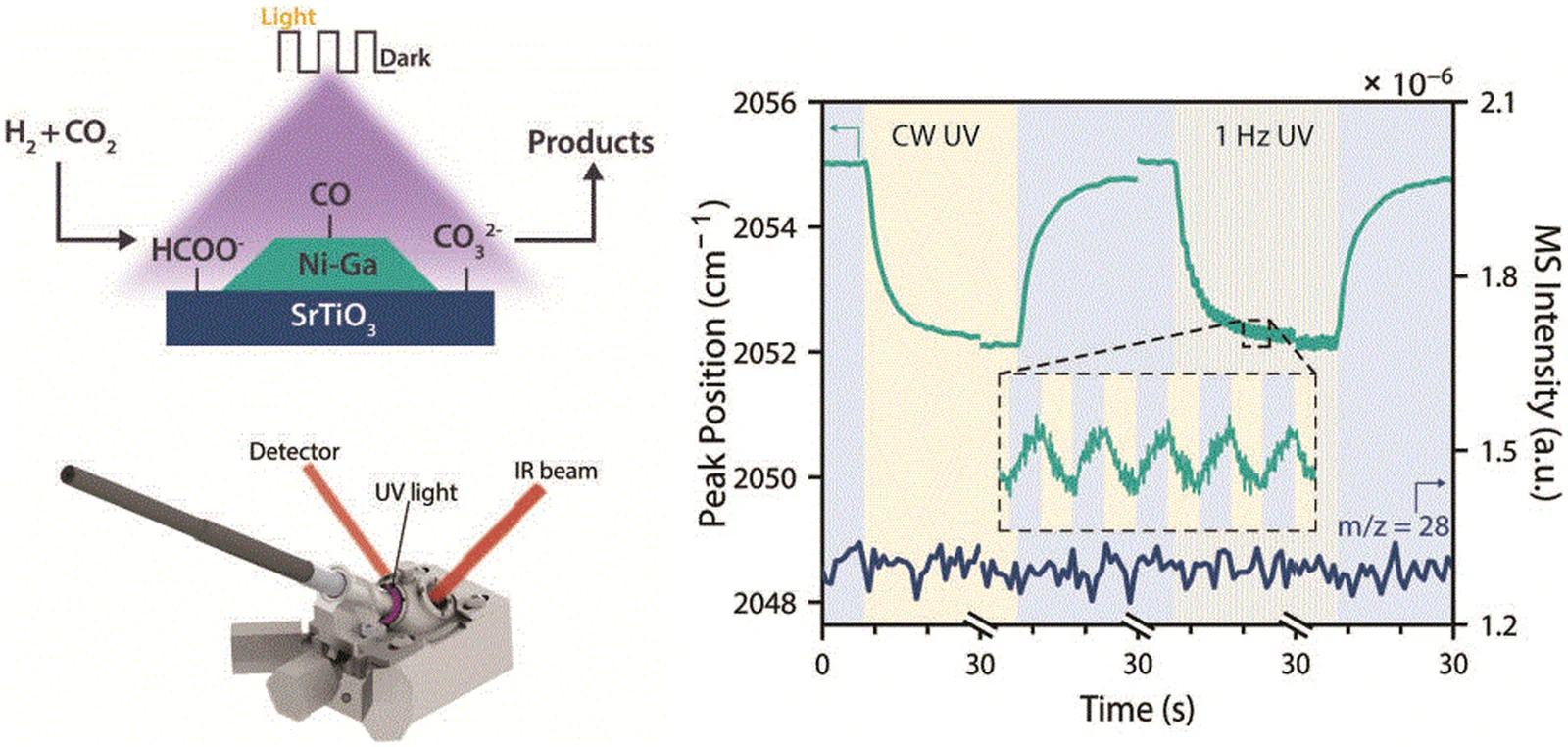

## Introduction

According to resonant catalysis (RC) theory, applying an intermittent stimulus on a heterogeneous catalyst can boost catalytic activity by three to four orders of magnitude and improve selectivity in parallel reactions from, *e.g.*, 50 to 100%.^1–4^ Theoretical RC models are based on the assumption that stimulation causes intermittent changes in the binding energies (BE) of adsorbed surface species, causing the catalyst to switch between surface-reaction-controlled and desorption-controlled regimes on a reaction’s volcano plot, and forming a catalytic ratchet.^5^ Most models predict that, in order to achieve resonant catalysis, the BE of reaction intermediates should change by at least 0.2 eV, at frequencies up to 10^5^ Hz.^3,6^ This introduces several challenges related to practically achieving intense and dynamic catalyst stimulation, and selecting the most effective stimulation parameters for a given catalyst and reaction.

In experimental settings, catalyst materials can be stimulated using charge and an electric field,^7^ light,^8^ heat^9^ or strain.^10^ Using light as a stimulus is advantageous because it is relatively easy to implement, can be pulsed at high frequencies, and its parameters, such as wavelength, intensity and duty cycle, can be readily adjusted.^5^ Continuous light was shown to improve the performance of catalytic hydrogenation of CO_2_ to CH_3_OH over commercial Cu–ZnO–Al_2_O_3_ catalysts by ∼25%,^11^ an effect attributed to electronic modification of the Cu–ZnO interface, which in turn favours the activation of formate species. Moreover, intermittent light (440 nm LED, 3.5 kHz, and 1.3 W cm^−2^) was shown to increase the rate of the decomposition of CH_3_OH into CO and H_2_ over Pt catalysts two-fold compared with continuous illumination.^8^ This was attributed to a decrease in CO coverage due to a lower BE of ∼0.25 eV under light irradiation, as determined by Temperature Programmed Desorption (TPD).^8^ However, more recent work indicates that light-induced CO desorption is not due to changes in the BE of adsorbed CO, but is instead photon-induced.^12^

These examples show that, while continuous and intermittent light can be used to stimulate catalyst materials, the observed enhancements in catalytic performance for intermittent light stimulation are far from the theoretically predicted range of orders-of-magnitude improvement. The discrepancy between RC theory and experiment may be due to several factors: (i) there are many mechanisms by which light can interact with catalysts and surface species, which depend on the chosen light for stimulation (wavelength, intensity, and duty cycle), catalyst formulation, nanostructure, and reaction conditions; and (ii) most theoretical models for resonant catalysis involve simple thought reactions of the type A to B, in which a certain effect of stimulation is assumed and treated implicitly, while real catalytic reactions consist of complex pathways and multiple intermediates. To bridge this gap, and to evaluate and guide the choice of stimulus parameters towards resonant catalysis, we need methods to monitor and study the effect of dynamic catalyst stimulation on catalyst surface chemistry and catalytic performance during stimulation and under reaction conditions.

Here, we demonstrate a method to study catalyst materials under dynamic stimulation, which we call “*stimulando*” spectroscopy. This terminology was recently introduced in a perspective article on the field of dynamic and stimulated catalysis,^5^ and it entails: (i) delivering the stimulus to the catalyst while acquiring spectroscopic data under reaction conditions, (ii) sampling the part of the catalyst material affected by the stimulus to observe changes in the catalyst and catalytic mechanism during stimulation, and (iii) monitoring the performance of the stimulated catalyst and correlating this with spectroscopic signatures to gain insights into effective catalyst stimulation. The method resembles that of well-established modulation excitation (ME) experiments, where the catalyst is exposed to periodically changing conditions, with two main differences. First, the ultimate purpose of *stimulando* experiments is to guide stimulus design, in addition to studying reactive species as in ME spectroscopy, and, secondly, light is used as the stimulation instead of varying gaseous concentration, as is common practice in ME and Steady-State Isotopic Kinetic Analysis (SSITKA)-DRIFTS.^13^ An example of how *stimulando* spectroscopy may guide stimulus design is by monitoring how fast surface species evolve upon intermittent stimulation, and changing the stimulation parameters to avoid reaching steady-state coverages during parts of the duty cycle, which would otherwise make the intermittent stimulation less effective.

**Fig. 1:**
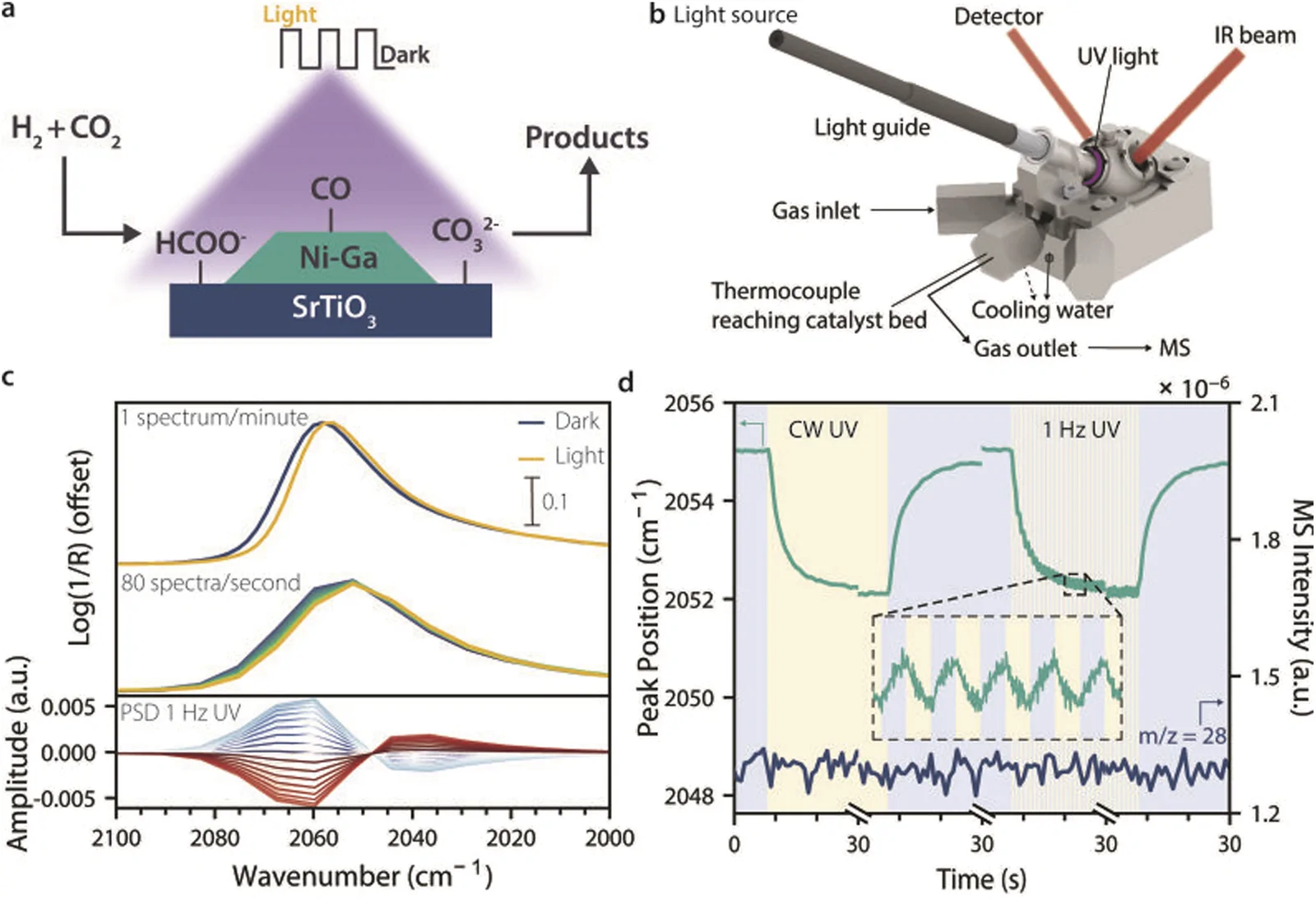
Concept of *stimulando* vibrational spectroscopy to study intermittent UV light effects on catalytic CO_2_ hydrogenation. (a) Ni_3_–Ga (4 wt%)/SrTiO_3_ catalysts were tested for light-assisted CO_2_ hydrogenation as a model reaction to probe the effect of intermittent light on the catalyst performance and surface chemistry. The most abundant surface species identified by infrared (IR) spectroscopy are shown (*i.e.*, CO, HCOO^−^, and CO_3_^2−^). (b) The catalyst was loaded into a commercial Harrick cell equipped with two ZnSe windows for diffuse reflectance infrared Fourier-transform spectroscopy (DRIFTS), and one quartz window fitted with an accessory for light-guide attachment for stimulation by ultraviolet (UV) light. The gas outlet was linked to a mass spectrometer (MS) for online performance analysis. (c) Spectral region of linearly adsorbed CO on Ni_3_–Ga/SrTiO_3_ upon switching on UV irradiation, shown as an example of light-induced changes in surface species. Transient changes in CO IR bands were too fast to be observed by steady-state DRIFTS (SS-DRIFTS, 1 spectrum min^−1^, top), but were followed by rapid-scan DRIFTS (RS-DRIFTS) with millisecond (ms) time resolution (80 spectra s^−1^, middle). To detect oscillations in surface coverages induced by intermittent UV light (1 Hz), we combined RS-DRIFTS with Phase Sensitive Detection (PSD) analysis (bottom). (d) CO IR peak-position shifts were tracked by RS-DRIFTS under continuous-wave (CW) and intermittent UV light (1 Hz), while reactants and products were followed by MS (shown: *m*/*z* = 28, CO). No change in catalytic performance was detected upon UV illumination despite the significant changes in surface species observed. Reaction conditions: (Ar or He) : H_2_ : CO_2_ = 20 : 15 : 5 mL min^−1^; *p* = 1 atm; *T* = 225 °C; UV light: 385 nm LED, 400 mW cm^−2^. For details on spectral processing, see Fig. S2 in the SI.

As a showcase of the method, we here used a 4 wt% Ni_3_–Ga/SrTiO_3_ catalyst to study intermittent light stimulation effects in CO_2_ hydrogenation with *stimulando* DRIFTS (Fig. 1a). The catalyst composition was chosen because Ni–Ga-based materials have been reported to produce methanol at ambient pressures, and because SrTiO_3_ is a semiconductor that can be excited with UV light and has previously been reported for light-assisted CO_2_ hydrogenation.^14–16^ A dedicated *stimulando* set-up and cell were built (Fig. 1b), and two DRIFTS methods, steady-state (SS-DRIFTS, 1 spectrum min^−1^, and 4 cm^−1^ resolution) and rapid-scan (RS-DRIFTS, 80 spectra s^−1^, and 16 cm^−1^ resolution), were used to observe how fast and to what extent surface species changed upon UV light illumination under CO_2_ hydrogenation conditions, as showcased in Fig. 1c for the spectral region of adsorbed CO species. Despite the lower wavenumber resolution for RS-DRIFTS, the peak shapes and positions were comparable between the two techniques, and the signal-to-noise ratio was only slightly lower for RS-DRIFTS. Moreover, PSD analysis was performed on the RS-DRIFTS spectra taken during intermittent UV light illumination to investigate changes during modulation and to check for the presence of short-lived species (Fig. 1c, bottom).

Using the technique of *stimulando* RS-DRIFTS, we were able to follow changes in the IR peak intensity and position, as well as their rates of change, for different surface species during the switch from dark to light and back at the millisecond (ms) timescale, while analysing the gas composition at the reactor outlet (Fig. 1d). We studied the effect of CW and intermittent light on different intermediates as a function of temperature, and showed that the intensity of the IR bands of CO, formate, and carbonate surface species decreased under illumination, and increased reversibly in the dark. This indicates that light caused a decrease in surface coverage of these species, due to inhibited formation from, *e.g.*, CO_2_, induced desorption, or faster reaction towards other species. The time to reach steady state was shorter for CO (on the order of seconds) than for formate and carbonate species (on the order of minutes), suggesting different mechanisms of light stimulation are at play. Moreover, despite the relatively modest irradiance and photon flux used herein, spectral changes were generally comparable (once a limit cycle was reached) or faster when switching UV light on than when switching to dark, indicating that relaxation of the catalyst surface in the dark is the rate-limiting step in light-stimulated catalysis. Short light pulses or extreme duty cycles (*e.g.* 0.1 : 99.9 light : dark) should therefore lead to more effective stimulation. While UV light irradiation induced significant changes in surface chemistry, it did not affect the rate of CO_2_ conversion to CO under the conditions used herein. The absence of correlation between *stimulando* DRIFTS and catalytic performance points to possible limitations in using this method to guide stimulus design, which will be evaluated in future studies using higher photon fluxes and catalyst materials that are more responsive to light.

## Experimental

### Chemicals and materials

Nickel(ii)nitrate hexahydrate (Ni(NO_3_)_2_·6H_2_O, 99.999% trace metals basis), gallium(iii)nitrate hydrate (Ga(NO_3_)_3_·*x*H_2_O, 99.999% trace metals basis), strontium titanate (SrTiO_3_, 99% trace metals basis, nanopowder <100 nm) and potassium bromide (KBr, ≥99% trace metals basis) were all purchased from Sigma-Aldrich and used without further purification.

### Catalyst material synthesis

A Ni–Ga/SrTiO_3_ catalyst was prepared by a double incipient wetness impregnation (IWI) synthesis method based on Studt *et al.*,^14^ aiming for a Ni : Ga molar ratio of 5 : 3 and 6 wt% total metal loading. The actual catalyst composition after synthesis was 4 wt% Ni_3_–Ga/SrTiO_3_, as determined by Inductively Coupled Plasma-Optical Emission Spectroscopy (ICP-OES), with a lower Ga loading than expected, which indicates that the Ga precursor adsorbed moisture during storage. The support was dried under vacuum overnight at 120 °C and impregnated with half of the metal nitrates solution in ultrapure water (UPW). The sample was again dried under vacuum for 4 h, after which a second impregnation was carried out. After impregnation, the sample was dried at 80 °C for 24 h. The sample was then ground and sieved to obtain a 75–125 µm size fraction. Finally, the sample was calcined in a plug flow reactor at 400 °C with a ramp of 5 °C min^−1^ for 4 h in compressed air and subsequently reduced at 700 °C with a ramp of 5 °C min^−1^ for 3 h in 30% H_2_/70% N_2_.

### *Ex situ* characterization techniques

Transmission electron microscopy (TEM) images were taken using a Tecnai T20 instrument operating at 200 kV. Energy-dispersive X-ray spectroscopy (EDX) elemental maps were obtained using a TFS Talos F200X instrument operating at 200 kV. X-ray diffraction (XRD) patterns were measured in Bragg mode on a Bruker D8 Advance instrument with a Cu source. ICP-OES analysis was carried out on a PerkinElmer Avio 500 spectrometer using LiBO_2_ digestion. UV-vis diffuse reflectance spectroscopy (UV-vis DRS) was carried out on a Lambda 950S UV-vis NIR spectrophotometer, which was equipped with a deuterium and halogen light source and an InGaAs detector. The samples were prepared for UV-vis DRS by diluting the powder samples with polytetrafluorethene (Teflon) powder to obtain sufficient reflectance counts. The diffuse reflectance values were related to absorption properties using the Kubelka–Munk (K–M) function. Temperature-programmed reduction (TPR) analysis was done on a 3P Altamira AMI-300ip high-throughput chemisorption analyser with a pretreatment drying step at 300 °C after which the samples were reduced in 5% H_2_/Ar up to 900 °C.

### *Stimulando* spectroscopy experiments

For *stimulando* DRIFTS experiments under light irradiation and CO_2_ hydrogenation conditions, we used a silica-coated commercial Harrick cell equipped with two ZnSe windows (2 mm thick, and 15 mm Ø) for IR light transmission, one quartz window (2 mm thick, and 15 mm Ø) for UV light transmission, heat-traced gas inlet and outlet, an internal thermocouple reaching the catalyst bed, and heating and water cooling for temperature control. The sample cup was first filled with quartz wool and subsequently with a silica-coated 400 × 400 mesh. On top of the mesh, ∼20 mg of catalyst sample was placed. The Harrick cell was put in a Harrick Praying Mantis™ diffuse reflection accessory, and a Bruker Tensor 37 FT-IR or Invenio-R spectrometer equipped with a mercury cadmium telluride (MCT) detector was used for the SS-DRIFTS and RS-DRIFTS measurements, respectively. SS-DRIFTS and RS-DRIFTS spectra were acquired every minute with 4 cm^−1^ resolution and every 12.5 ms with 16 cm^−1^ resolution, respectively. Spectra collected from dried KBr powder (80 °C) at room temperature under He or Ar flow were used as the *I*_0_ reference. A gas chromatograph (GC, Interscience, custom-built Global Analyzer Solutions (G.A.S.) Compact GC_4.0_) or a mass spectrometer (MS, GSD 350 Omnistar Pfeiffer) was used for online gas analysis for the SS- and RS-DRIFTS measurements, respectively. A Thorlabs Chrolis C2 instrument equipped with six different LEDs (385, 420, 475, 565, 590, and 625 nm) was used as the light source. In this study, we used a 385 nm LED set at 400 mW cm^−2^ irradiance and 7.8 × 10^17^ photons cm^−2^ s^−1^ photon flux. A light guide was used to deliver light to the sample and was equipped with a MgF_2_-coated aspheric condenser lens (Edmund Optics) to focus the light beam into an elliptical spot of 1 cm^2^ on the sample. A home-built accessory was attached to the dome of the Harrick cell to hold the light guide perpendicular to the quartz window at a distance of 2 cm from the sample. In a typical experiment, the Ni_3_–Ga/SrTiO_3_ catalyst was first reduced at 400 °C for 1 h in 50% H_2_/Ar or He (20 : 20 mL min^−1^) with a temperature ramp of 5 °C min^−1^ (based on TPR results, Fig. S1). After this, the catalyst was cooled to 200 °C, at which point the gas feed was switched to the reaction gases: (Ar or He) : H_2_ : CO_2_ = 20 : 15 : 5 mL min^−1^, 1 atm total pressure. The temperature was increased to different reaction temperatures of 200, 225, 250, and 275 °C. At every reaction temperature, the catalyst was exposed to dark, continuous-wave (CW), and intermittent UV illumination (50% duty cycle, 1 Hz frequency), using the same average irradiance and photon flux for CW and intermittent UV illumination. An IR camera (A700 from FLIR) was used to record the catalyst surface temperature increase due to both CW and 1 Hz illumination at each temperature during light-stimulated CO_2_ hydrogenation.

## Results and discussion

### Monitoring surface species during UV-light-stimulated catalysis as a function of temperature

A 4 wt% Ni_3_–Ga catalyst on a SrTiO_3_ support was prepared by a double incipient wetness impregnation method based on Studt *et al.*^14^ Details on the characterization of the catalyst can be found in Fig. S1. EDX elemental mapping showed that Ni and Ga formed bimetallic particles, with some Ga segregation on the support itself. TEM showed that the synthesized Ni_3_–Ga nanoparticles were 5–12 nm in size, with an average of size of 8 ± 1.5 nm. The light absorption of the catalyst materials was tested by UV-vis DRS, which revealed a broad band below 380 nm with an absorption edge at 390 nm, corresponding to a bandgap of 3.2 eV that is characteristic of SrTiO_3_.^17^ Accordingly, 385 nm UV light was used for the stimulation.

**Fig. 2:**
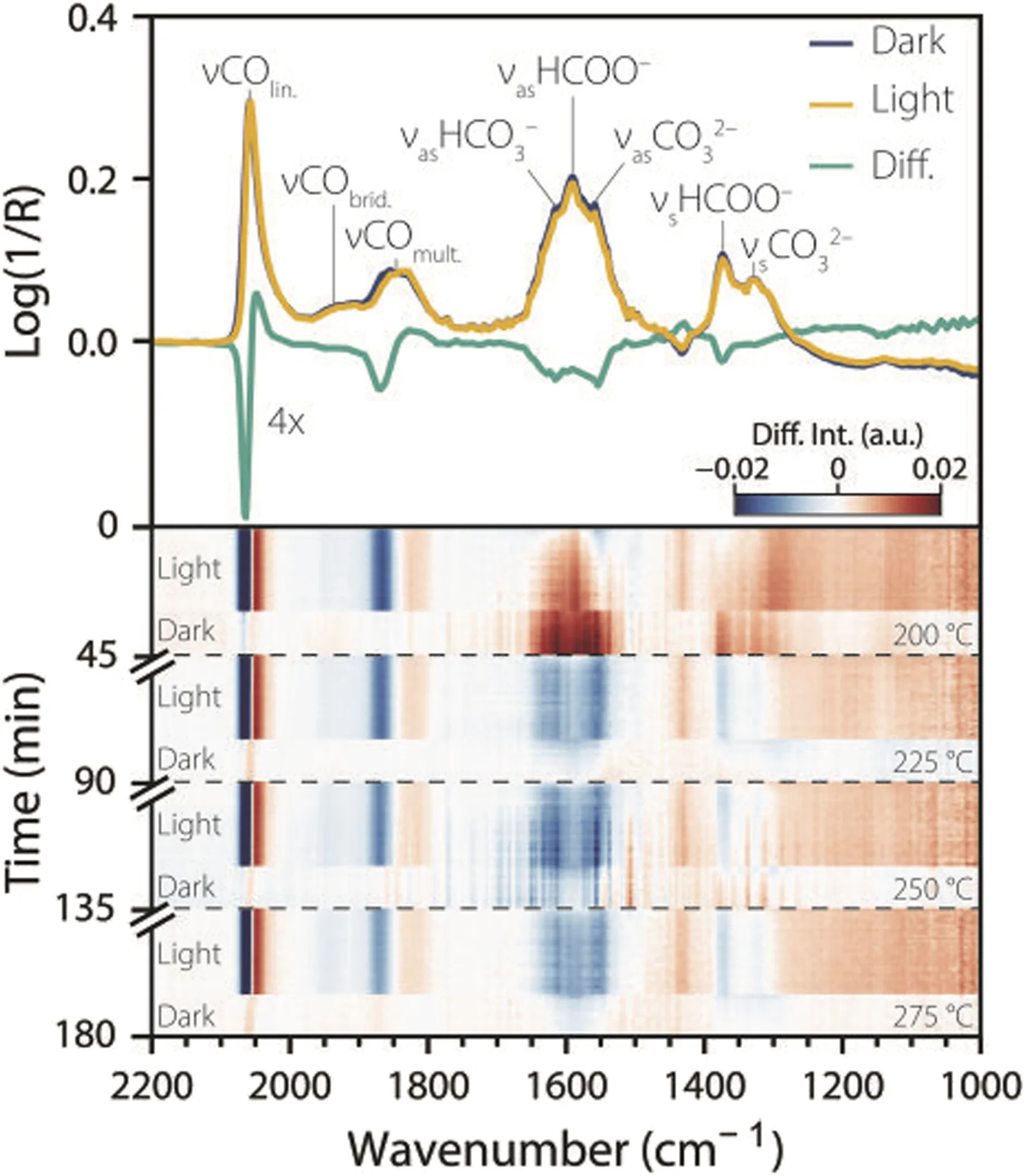
Temperature dependence of UV light effects on surface species during catalytic CO_2_ hydrogenation. Top: two consecutive steady-state diffuse reflectance infrared Fourier-transform spectroscopy (SS-DRIFTS) spectra taken at 225 °C under CO_2_ hydrogenation conditions under dark (blue line) and UV light (yellow line) conditions, showing multiple IR peaks with their corresponding assignments. A difference spectrum (light minus dark) is shown in teal, scaled by a factor of four. Bottom: heatmap showing UV-light-induced spectral changes over time and temperature. At each temperature, a SS*-*DRIFTS spectrum recorded before turning on the UV light was subtracted from all subsequent spectra, including the 30 min light period and the 15 min dark period after illumination. Reaction conditions: He : H_2_ : CO_2_ = 20 : 15 : 5 mL min^−1^; *p* = 1 atm; UV light: 385 nm LED, 400 mW cm^−2^.

The pre-reduced and calcined Ni_3_–Ga/SrTiO_3_ catalyst was reduced *in situ* in a Harrick cell at 400 °C, and tested for CO_2_ hydrogenation under dark, CW, and intermittent UV light irradiation at different temperatures (200–275 °C), while acquiring *stimulando* DRIFTS spectra (Fig. 2). The top panel of Fig. 2 shows two consecutive *stimulando* SS-DRIFTS spectra under dark and UV light conditions at 225 °C, and their difference spectrum (light–dark) multiplied by a factor of four to highlight spectral changes. The spectra were baseline-corrected by subtracting a spectrum acquired during reduction at 400 °C, aligned, and further corrected by subtracting the water rovibrational bands (Fig. S2). Under dark conditions, a sharp, intense IR peak was observed at ∼2058 cm^−1^, which can be attributed to linearly adsorbed CO on nickel.^18^ This peak was followed by a shoulder at ∼1925 cm^−1^ and a broad peak at ∼1845 cm^−1^, both with lower intensities, which can be attributed to bridged- and multi-bound CO on nickel, respectively.^18^ A broad peak with high intensity was observed at ∼1590 cm^−1^, which we assign to the anti-symmetric stretch of adsorbed bidentate formate.^11,19,20^ This broad peak presented shoulders at ∼1615 and ∼1558 cm^−1^ corresponding to the anti-symmetric stretch of bidentate bicarbonate and carbonate, respectively.^11,19–21^ Lastly, two IR peaks with moderate width and height were observed at ∼1372 and ∼1325 cm^−1^ which can be attributed to the symmetric stretch of bidentate formate^11,19,20^ and the symmetric stretch of monodentate carbonate, respectively.^20–22^ Rasteiro *et al.* also observed the three orientations of adsorbed CO and formate bands during CO_2_ hydrogenation at 225 °C and ambient pressure on an unsupported Ni_5_–Ga_3_ catalyst, but no carbonate bands were observed in their case.^23^ Wei *et al.* observed similar formate and carbonate species at 220 °C and atmospheric pressure for a Cu/Mn–SrTiO_3_ catalyst.^24^ Both these studies also reported adsorbed methoxy and methanol species in the 1100–1000 cm^−1^ spectral range, which were not observed in our work. This is consistent with the (unfortunate) sluggish activity of the synthesized Ni_3_–Ga/SrTiO_3_ catalyst for CO_2_ hydrogenation, as also reported in literature for this ratio,^14,25^ and the formation of CO *via* the reverse water–gas shift (rWGS, with a CO_2_ conversion in the range of 3%, 25 mmol CO g^−1^ Ni_3_–Ga).

Upon switching from dark to CW UV illumination, the linear CO band decreased in intensity and shifted to lower wavenumber, resulting in negative and positive features in the difference spectra at ∼2067 and ∼2049 cm^−1^, respectively. Similarly, the IR peak intensity of multi-bound CO decreased at ∼1871 cm^−1^ and increased slightly at ∼1829 cm^−1^. Comparable redshifts in peak position upon illumination have been reported in the literature for linearly adsorbed CO on Pt nanoparticles and were attributed to photon-induced CO desorption.^12^ On the other hand, the bridged CO IR peak was almost unaffected by UV light, indicating that certain CO species are less prone to desorption, despite the seemingly similar adsorbate structures. An alternative explanation for the observed changes in CO peaks upon irradiation can be given based on the model of CO vibrational coupling: at high coverages of CO, vibrational coupling can induce the formation of collective, normal vibrational modes of ensemble of CO molecules.^26^ A decrease in intensity and a shift to lower wavenumbers indicate that vibrational coupling was partially disrupted by light, due to a decrease of CO coverage, the breaking of CO adsorbate islands, and/or changes in dipole moment due to light excitation of CO. In both interpretations, the results indicate a decrease in CO coverage upon UV light irradiation.

The IR peaks at ∼1615, ∼1558 and ∼1372 cm^−1^ decreased in intensity upon UV irradiation, pointing to a decrease in surface coverage of bidentate formates and (bidentate) carbonates. These observations are consistent with Boga *et al.*, who reported a decrease in intensity upon illumination for the IR peaks at ∼1644, ∼1587 and ∼1370 cm^−1^ on NiO/(SrTiO_3_/SrCO_3_) at room temperature,^15^ and with Xie *et al.* who observed an intensity decrease for the IR band for formate species located at ∼1372 cm^−1^.^11^ On the other hand, the IR peaks at ∼1590 and ∼1325 cm^−1^ were unaffected by UV illumination, which was not observed in the previously mentioned literature, indicating that some formate and carbonate species are less responsive to light than others. Moreover, an IR peak at ∼1431 cm^−1^ increased in intensity upon UV light illumination, and decreased in the dark. This feature is attributed to the symmetric stretch of polydentate carbonate,^19,21^ and it appears as a negative band because it was present in the subtracted reduction spectrum at 400 °C (Fig. S2). This indicates that carbonate species are strongly bound to the catalyst and are spectator species in the reaction, as previously reported for similar catalysts.^15^

The bottom panel of Fig. 2 shows a heatmap of the subtracted spectra (light–dark) over time for different reaction temperatures. For each temperature, the last spectrum in the dark before turning on the light was used for the subtraction to reveal changes with respect to the dark. The main spectral changes induced by light were observed at comparable wavenumbers, regardless of temperature. However, the extent to which IR bands changed in intensity was dependent on the temperature and the type of surface species. For example, while the linearly adsorbed CO showed a comparable response to UV light at different temperatures, the multi-bound CO was less responsive at higher temperatures, as indicated by the fading of the blue colour at ∼1871 cm^−1^. The IR peak assigned to bicarbonate species at ∼1615 cm^−1^ started to increase in intensity upon illumination at 200 °C, but it decreased in intensity at higher temperatures, with a maximum decrease due to light at 250 °C. The IR peak of formate species at ∼1590 cm^−1^ increased in intensity upon UV irradiation at 200 °C, but its intensity remained relatively unchanged at higher temperatures. These observations show that different surface species are more responsive towards light stimulation under different conditions, and showcase the type of insights that can be gained using *stimulando* spectroscopy and the complementarity of the method with respect to probe-molecule approaches.

**Fig. 3:**
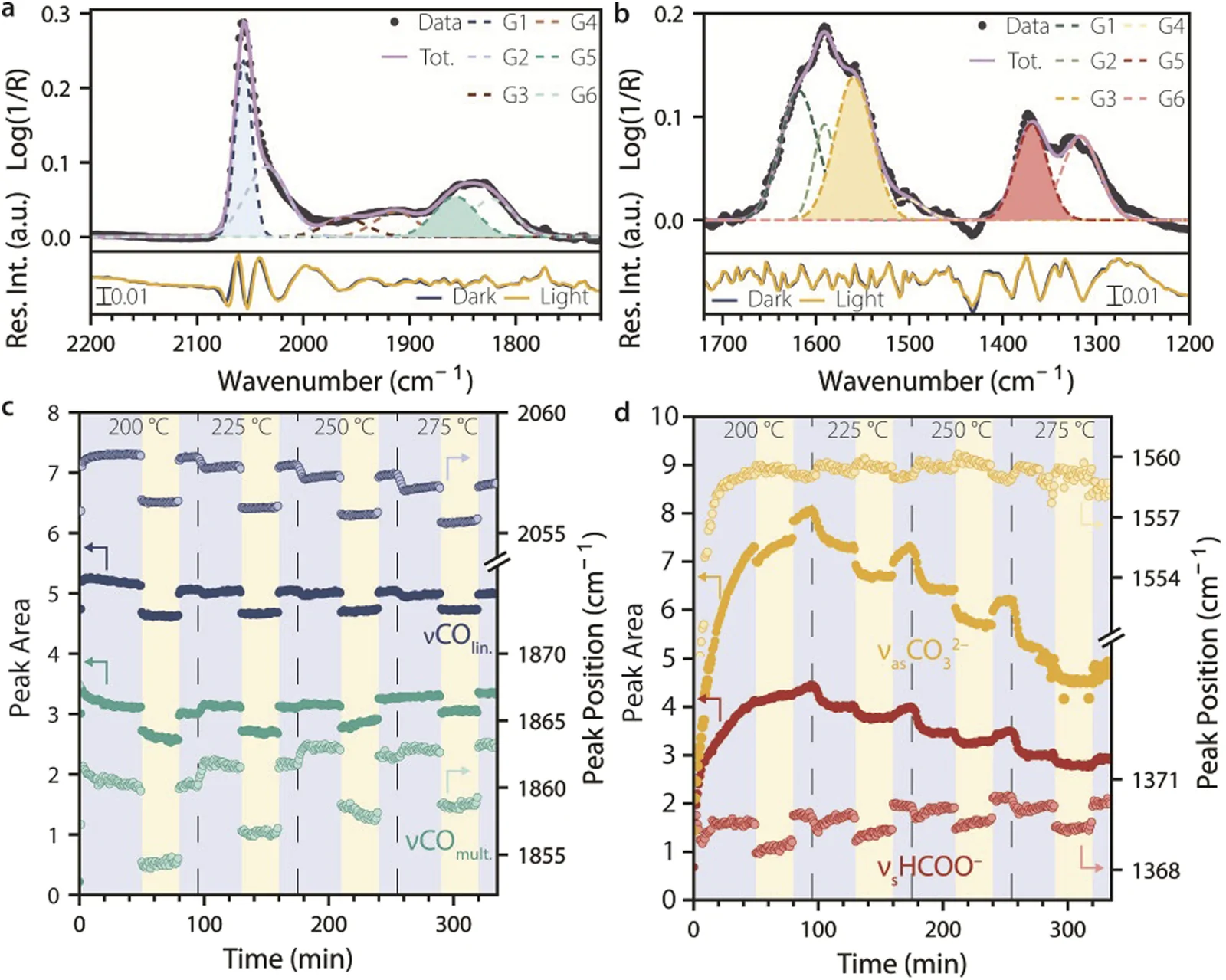
Tracking changes in surface species during catalytic CO_2_ hydrogenation under dark and UV illumination conditions. (a and b) Top: steady-state diffuse reflectance infrared Fourier-transform spectroscopy (SS-DRIFTS) spectra taken during CO_2_ hydrogenation at 225 °C under dark conditions showing (a) the adsorbed CO region (2200–1720 cm^−1^) and (b) the formate and carbonate region (1720–1200 cm^−1^), and the respective Gaussian fitting functions. Bottom: representative residuals from the Gaussian fit under both dark and UV light conditions. Six functions were chosen to minimize the residuals of the fit. (c and d) Gaussian-fitted peak area (solid symbols) and peak position (empty symbols) over time during dark and UV light periods, at different reaction temperatures, for the following IR bands: (c) linearly adsorbed CO (*ν*CO_lin._, 2055–2060 cm^−1^), multi-bound CO (*ν*CO_mult._, 1855–1865 cm^−1^), (d) carbonate species (*ν*_as_CO_3_^2−^, 1557–1560 cm^−1^), and formate species (*ν*_s_HCOO^−^, 1368–1371 cm^−1^). Reaction conditions: He : H_2_ : CO_2_ = 20 : 15 : 5 mL min^−1^; *p* = 1 atm; UV light: 385 nm LED, 400 mW cm^−2^.

To quantitatively evaluate the effect of UV light on different surface species as a function of temperature, the CO (2200–1720 cm^−1^, Fig. 3a and c) and formate/carbonate regions (1720–1200 cm^−1^, Fig. 3b and d) of the SS-DRIFTS spectra were fitted with a set of six Gaussian functions each, to obtain a residual of <±0.02 pseudo-absorbance units (bottom panels in Fig. 3a and b). The four Gaussian functions that showed the most significant changes as a function of UV light irradiation corresponded to peaks attributed to linear CO, multi-bound CO, formate (HCOO^−^) and carbonate (CO_3_^2−^) species (shaded Gaussians in Fig. 3a and b). The changes in peak area and peak position for these fitting functions were plotted as a function of time in Fig. 3c and d for a CO_2_ hydrogenation experiment where the catalyst material was heated to different reaction temperatures (200, 225, 250, and 275 °C) and exposed to a dark period, a UV light period and then again to a dark period at each temperature. The peak intensity showed comparable trends to those of the peak area (Fig. S3).

Fig. 3c looks at the peak intensities and positions for linearly and multi-bound adsorbed CO. For both species, the peak areas and peak positions decreased when the light was switched on and increased again reversibly when the light was switched off. The peak area and position remained overall constant during the dark and light periods (except for CO_mult._ at 250 °C), implying that steady state was reached within a minute under both UV light irradiation and dark conditions (*i.e.*, faster than the time resolution of the SS-DRIFTS spectra). Notably, the CO IR peak area and peak position under dark conditions slightly decreased with increasing temperature for the CO_lin._ species, while they increased with increasing temperature for the CO_mult._ species. On the other hand, the extent of the change in the peak area decreased with temperature for both species, from 9.6 to 5.0% for CO_lin._ and from 9.7 to 7.6% for CO_mult._ at 200 and 275 °C, respectively. The change in peak position (Δ*ν*) remained roughly constant for CO_lin._ (Δ*ν* ∼2 cm^−1^), while it decreased for CO_mult._ from 6 to 4 cm^−1^ at 225 *vs.* 275 °C.

The observed changes in the CO IR peaks upon light irradiation cannot be explained in terms of light-induced heating, as demonstrated by an IR heat camera (Fig. S4 and S5). While light induced a change in catalyst surface temperature of 30 °C, the light-induced changes in the CO IR spectra were more significant than temperature-induced changes observed upon heating under dark conditions by 75 °C. Changes in the peak position and peak area of the stretching mode of adsorbed CO molecules are widely interpreted in the literature as (i) a change in CO BE, with a peak position at lower wavenumber indicating a weaker C–O bond compared to a peak position at higher wavenumber, in turn suggesting that the remaining adsorbates have a stronger metal–CO bond, and (ii) a change in the coverage of CO on the catalytic surface, assuming that peak area is proportional to coverage, and that vibrational-coupling effects among CO molecules are negligible. Under these assumptions, our results indicate that higher reaction temperatures lead to a decrease in CO_lin._ coverage and increasing CO_mult._, while light causes a decrease in CO coverage for both species, to a lesser extent at higher temperatures. If we assume that vibrational-coupling effects are at play, the changes in the CO IR peak area and peak position cannot be directly correlated to CO coverage and CO BE.^26^ Nonetheless, according to this model, the observed changes are consistent with a decrease in CO surface coverage due to UV light irradiation.

While our observations are consistent with previously reported light-induced CO desorption on Pt catalysts,^8^ no change in CO concentration were detected by online GC analysis during light irradiation (Fig. S6). This suggests that a light-induced decrease in CO coverage may be due to other phenomena, such as a hindered formation of adsorbed CO from CO_2_ dissociative adsorption, or light-induced conversion of CO to other adsorbed species. This deserves further attention in future studies, for example, using CO_2_ as a probe molecule in TPD and temperature-programmed surface reaction (TPSR) experiments.

Fig. 3d shows the peak area and peak position of the two fitted Gaussian functions for CO_3_^2−^ and HCOO^−^ species. For both species, the peak areas decreased by 5–6% under UV light, but they slowly increased at 200 °C and then decreased at higher temperatures, during dark and light periods alike. This indicates that formate and carbonate are strongly bound to the catalyst surface, with a tendency to accumulate at low temperatures, and requiring higher temperatures to be removed. In contrast with what was observed for CO species, the peak areas changed slowly when switching the UV light on and off, reaching steady state in the order of minutes. The peak positions of the formate IR band showed a similar trend compared to that of CO_mult._, with the peak position decreasing with light but increasing with temperature. However, the peak shift was smaller compared to that of multi-bound CO (Δ*ν* = 0.5–1 cm^−1^). The peak position of the carbonate species was even less affected by light, but showed a slight increase during light and also with increasing temperature. The comparable effect of UV light and of a temperature increase of 30 °C under dark conditions strongly suggests that the changes in formate and carbonate species are due to heat effects induced by irradiation, based on IR heat camera measurements.

### Following dynamic changes in surface chemistry upon UV light switching

**Fig. 4:**
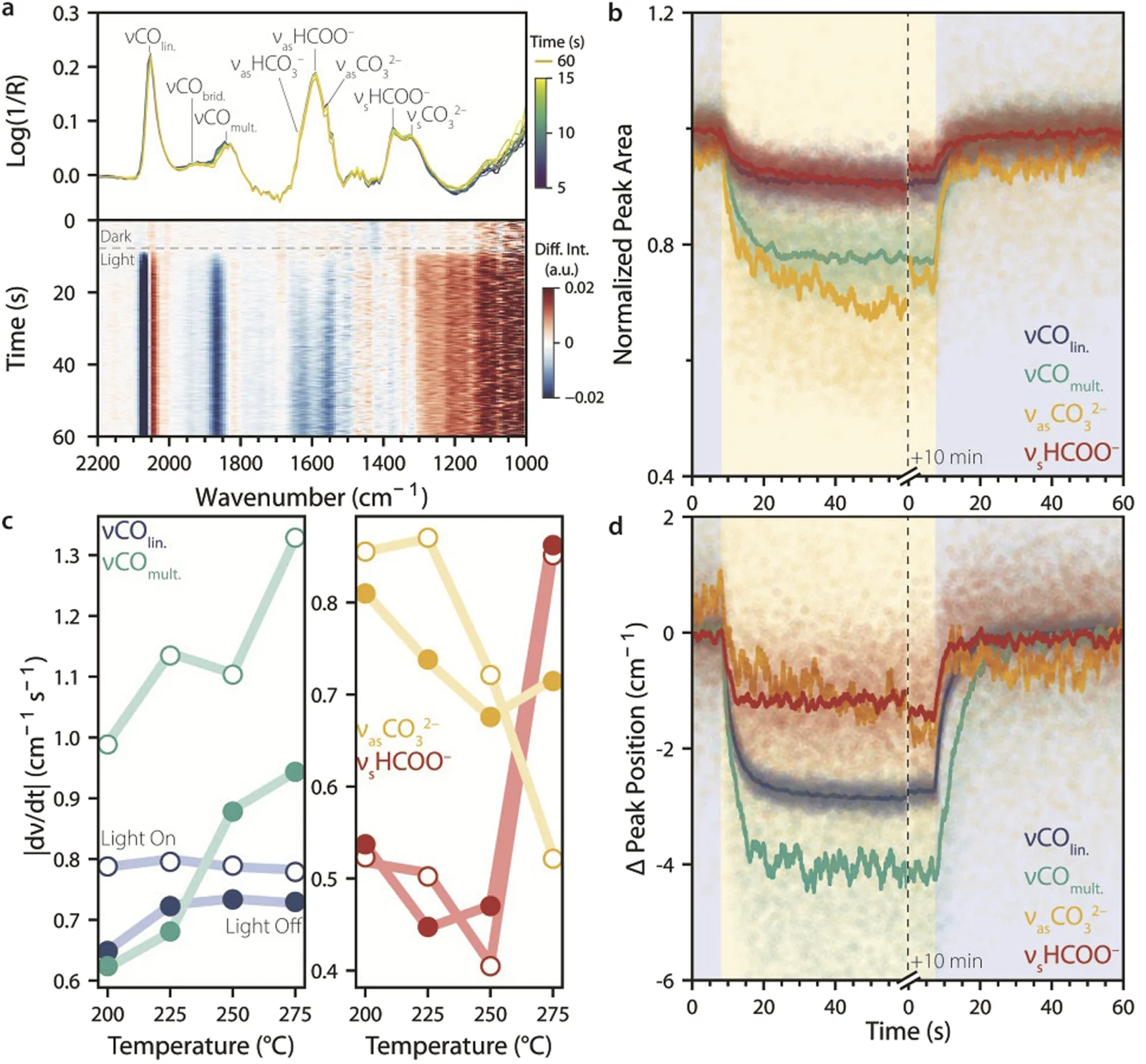
Following the evolution of surface species upon UV light switching during catalytic CO_2_ hydrogenation. (a) Top: rapid-scan diffuse reflectance infrared Fourier-transform spectroscopy (RS-DRIFTS) spectra taken under CO_2_ hydrogenation conditions at 225 °C over 10 s when switching from dark to light conditions (*t* = 5 s), with 12.5 millisecond (ms) time resolution, and their corresponding band assignments, showing fast and slight changes in band intensity and position. A spectrum taken after 60 s is also shown. Bottom: heatmap showing light-induced spectral changes over time, obtained by subtracting the spectrum measured under dark conditions (*t* = 0 s). (b and d) Change in (b) normalized peak area and (d) peak position upon switching from dark-to-light or light-to-dark (shaded areas: blue for dark, and yellow for light) at 225 °C, obtained from Gaussian fitting as in Fig. 2 (symbols: time-resolved data, lines: data running average, *n* = 100). The fitted IR peaks are assigned as follows: linearly adsorbed CO (*ν*CO_lin._, 2055–2060 cm^−1^), multi-bound CO (*ν*CO_mult._, 1855–1865 cm^−1^), carbonate species (*ν*_as_CO_3_^2−^, 1557–1560 cm^−1^), and formate species (*ν*_s_HCOO^−^, 1368–1371 cm^−1^). (c) Rate of change in peak position upon switching from dark-to-light (empty symbols) or light-to-dark (solid symbols) for the different intermediates as a function of temperature, obtained from the maximum values of the averaged derivative of the running-averaged peak positions shown in panel (d). Shifts in peak positions were generally faster when the light was switched on than when it was turned off, with the exception of CO_3_^2−^ and HCOO^−^ species at high temperature. Reaction conditions: Ar : H_2_ : CO_2_ = 20 : 15 : 5 mL min^−1^; *p* = 1 atm; UV light: 385 nm LED, 400 mW cm^−2^.

We have shown that the surface species adsorbed on Ni_3_–Ga/SrTiO_3_ catalysts during CO_2_ hydrogenation reach steady state in less than a minute when switching UV light on or off. To study how fast the system evolves towards steady state upon light switching at different temperatures, we used RS-DRIFTS (80 spectra s^−1^). Fig. 4a shows an example of RS-DRIFTS spectra taken over 10 s when switching on UV light at 225 °C, along with the corresponding peak assignments. Despite the lower spectral resolution (16 cm^−1^*vs.* 4 cm^−1^ for SS-DRIFTS) the RS-DRIFTS spectra and spectral changes induced by UV light were qualitatively comparable with what was observed with SS-DRIFTS (Fig. 2). However, the gradual evolution of surface chemistry could now be tracked on the 10 ms timescale, as shown in the heatmap of subtracted spectra (light–dark, Fig. 4a, bottom panel). This revealed that CO_lin._ reached steady state the fastest, followed by CO_mult._, and then (bi)carbonate and formate species. No additional short-lived species were observed compared with SS-DRIFTS.

To quantitatively measure the rate and extent of change in IR bands upon switching from dark-to-light and light-to-dark, the RS-DRIFTS spectra were fitted with the same Gaussian functions used for SS-DRIFTS in Fig. 3. Fig. 4b and d show the normalized peak areas and changes in peak position over time during dark–light–dark switching for the four Gaussians corresponding to the species most responsive to light (CO_lin._, CO_mult._, CO_3_^2−^, HCOO^−^). Since a total of 9600 spectra were fitted (120 s at 80 spectra s^−1^), the running average (*n* = 100) for the peak area and Δ*ν* are also shown for clarity. The normalized peak area changed the most (∼20%) for the carbonate and multi-bound CO species, and the largest peak-position change was observed for absorbed CO species (Δ*ν* = 2–4 cm^−1^). Upon switching on UV light, the linear and multi-bound CO species reached steady state within ∼11 and 16 s, respectively, and their peak areas did not change significantly after the 10 min light period. On the other hand, the carbonate and formate species did not reach steady state within 1 min.

Generally, the IR peak areas and positions changed more slowly when switching the light off than when switching the light on, as shown by the initial (fastest) rate of change in peak position during switching (Fig. 4c). The initial rate of change of the IR peaks also depended on temperature, in different ways depending on the species: for linearly adsorbed CO, the rate was fairly constant with temperature, while for multi-bound CO it increased with temperature. For carbonate species, the rate of change was higher upon switching from dark to light than from light to dark, except at 275 °C. On the other hand, for formate species, the light-on and light-off rates of change were comparable across the whole temperature range, increasing suddenly at 275 °C. While the reasons for the temperature dependence of the rate of change in IR peak positions are unclear, faster surface equilibration is expected at higher temperatures due to faster adsorption, desorption, surface diffusion, and reaction rates. Overall, these results show that changes in catalyst surface chemistry induced by light under these conditions occur on the order of seconds, and that relaxation in the dark is, on average, slower. This has implications for the choice of frequency, intensity, and duty cycle for dynamic catalyst stimulation by intermittent light, which we evaluated next.

### Tracking intermittent UV light effects on catalyst surface chemistry

**Fig. 5:**
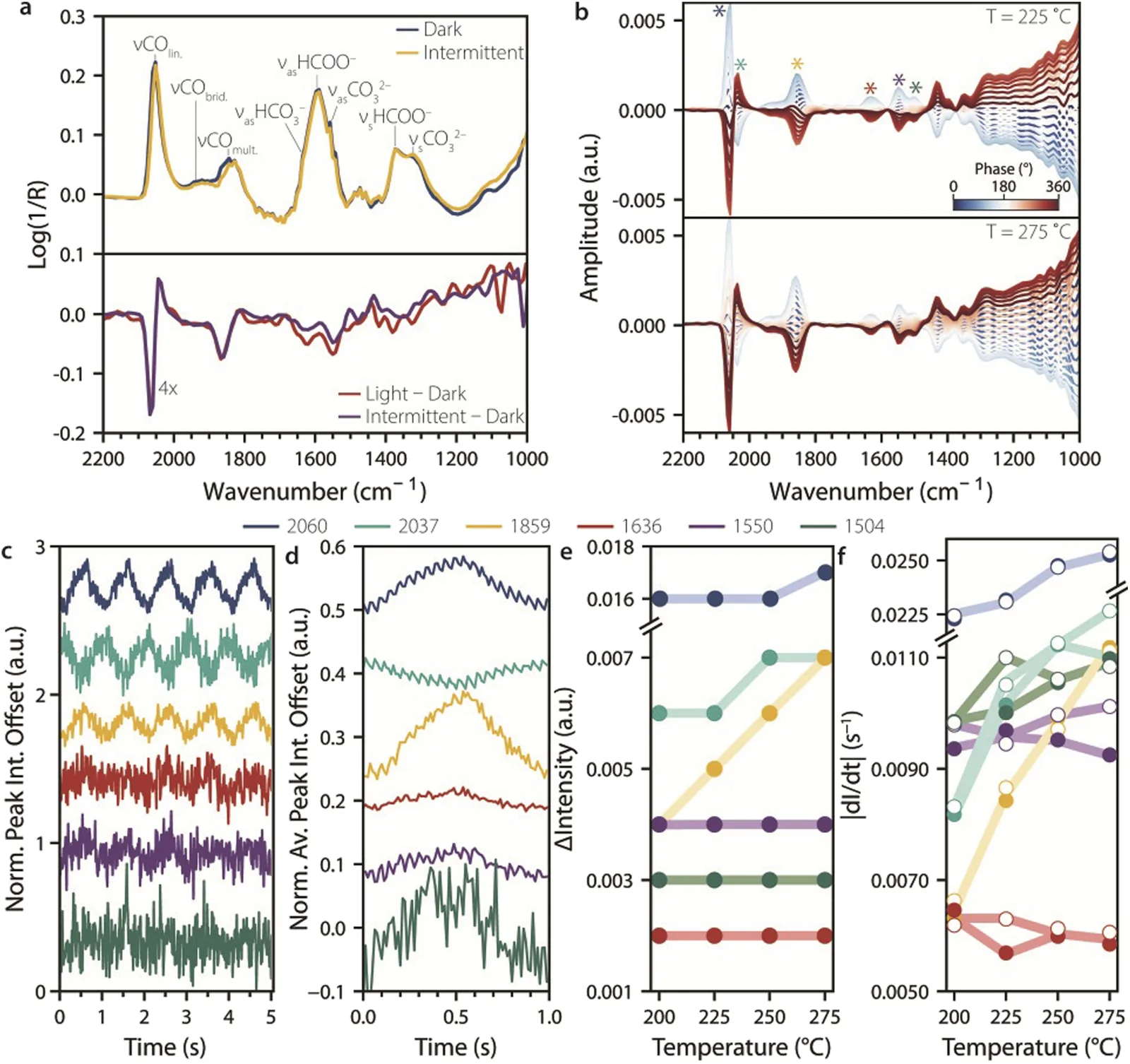
Tracking intermittent UV light effects on surface species during catalytic CO_2_ hydrogenation. (a) Top: rapid-scan diffuse reflectance infrared Fourier-transform spectroscopy (RS-DRIFTS) spectra recorded under CO_2_ reaction conditions during dark and intermittent UV light (averaged over 15 duty cycles) at 225 °C, with their corresponding IR band assignments. Bottom: difference spectra obtained by subtracting the dark spectrum from the spectra recorded under continuous (red line) and averaged intermittent illumination (purple line), showing that IR spectra under intermittent and continuous UV illumination are comparable. (b) Phase-Sensitive Detection (PSD) analysis of RS-DRIFTS spectra measured during intermittent UV light at 225 and 275 °C, highlighting the presence of six peaks within the wavenumber range not affected by background shift (asterisks). (c and d) Normalized and offset IR peak intensities of the six detected peaks at 225 °C, (c) during five seconds of intermittent light oscillations and (d) averaged over 30 light on/off cycles. (e) Change in intensity of the averaged oscillations calculated in (d) for each temperature. (f) Running-averaged (*n* = 50) derivatives of the peak intensity over time, showing the rate of change in peak intensity for light-to-dark (filled symbols) and dark-to-light (open symbols). Reaction conditions: Ar : H_2_ : CO_2_ = 20 : 15 : 5 mL min^−1^; *p* = 1 atm; UV light: 385 nm LED, 400 mW cm^−2^.

To study the effect of intermittent UV light stimulation on the catalyst material, we used 1 Hz intermittent light at a 50% duty cycle. This initial choice of stimulation frequency was based on the *stimulando* RS-DRIFTS results in Fig. 4, which showed spectral changes occurring on the timescale of seconds. Fig. 5a shows the (averaged) RS-DRIFTS spectra taken at 225 °C during a dark and intermittent light period of 15 s (top), and the difference spectra between dark and continuous light compared to dark and intermittent light (bottom). The difference spectra are comparable, indicating that the catalyst surface species during intermittent light irradiation resemble those under CW light irradiation. This is in accordance with the higher rate of change in the IR spectra observed when switching the light on than when switching the light off (Fig. 4c). Using higher frequencies for intermittent light stimulation (*e.g.*, 10 Hz) produced behaviour similar to continuous illumination, as the system remains in a state similar to the light-on state. This is consistent with a faster light-on rate of change compared to the light-off rate observed for most intermediates. In contrast, lower frequencies (*e.g.*, 0.1 Hz) induced changes similar to those observed at 1 Hz, but over a longer timescale (Fig. S7).

Since the RS-DRIFTS spectra were collected during intermittent light with a frequency much slower than the spectral sampling rate, the experiment can be treated as a ME experiment in which light serves as the modulation.^27^ Accordingly, we run PSD analysis on the RS-DRIFTS spectra to filter out random noise and to track subtle spectral changes induced by light stimulation,^28^ such as those due to the formation of short-lived species upon switching on or off UV light. Six peaks were identified by PSD on RS-DRIFTS spectra recorded during 30 cycles of 1 Hz UV light at 225 °C and 275 °C (Fig. 5b, asterisks), at positions comparable to the observed spectral changes in dark–light difference spectra (∼2060, ∼2037, ∼1859, ∼1636, ∼1550 and ∼1504 cm^−1^).

The normalized peak intensity over time at these wavenumbers during five duty cycles at 225 °C is shown in Fig. 5c. Oscillation in intensity can be seen at ∼2060, ∼2037, and ∼1859 cm^−1^, corresponding to CO peaks, and less clearly at ∼1636, ∼1550, and ∼1504 cm^−1^, for formate and carbonate species, respectively. This is expected based on the relatively slow response to light stimulation for these species compared to CO. It was observed that the 2037 cm^−1^ signal intensity correlates with the redshift of the linearly adsorbed CO band upon irradiation, and is therefore in opposite phase compared to the other wavenumbers. To improve signal-to-noise, Fig. 5d shows the change in intensity at the selected wavenumbers averaged over 30 duty cycles. In addition to the 1 Hz component arising from intermittent light stimulation, other frequencies in the IR signal response over time were detected at 3, 5 and 20 Hz by Fourier-transform analysis of the IR peak intensity of CO_lin._ (Fig. S7). The 3 and 5 Hz components reflect the fact that the oscillation in IR signal is not purely sinusoidal, and they are expected for a box function perturbation such as the one used herein, which can be approximated by a sum of odd harmonic sinusoidal functions. Accordingly, when using 0.1 Hz intermittent light, odd harmonics with decreasing contributions were observed (*e.g.* 0.3, 0.5, 0.7, and 0.9 Hz, Fig. S7, top). On the other hand, the 20 Hz signal frequency component is unexpected, and was observed for 0.1, 1, and 10 Hz intermittent light stimulation alike (Fig. S7). This suggests that the frequency is not related to the stimulation, and we note that it corresponds to that of the scanning of the interferometer mirror during RS-DRIFTS spectra acquisition, suggesting that the fast oscillation may be an artefact related to rapid-scan operation.

To quantify the extent of light-induced spectral changes at the different wavenumbers, we plotted the difference in intensity (ΔIntensity) between maxima and minima of the oscillations for the different wavenumbers at different temperatures (Fig. 5e). The ΔIntensity values for the CO peaks were the largest, and the extent of change increased with increasing temperature, while other species showed lower ΔIntensity values that were not sensitive to temperature. Adsorbed CO was therefore more affected by light than the other species, both under continuous and intermittent light simulation. Finally, we calculated the derivative for the change in intensity over time, to gain information about the rate of change during intermittent light stimulation (Fig. 5f). This shows that the catalyst responded at a similar rate to the light switching on or off, which is different from what has previously been observed for CW light. This difference may be explained by the fact that when a system is dynamically stimulated, it approaches a limit cycle, where surface species still change with each oscillation but at a different rate than at the start of the stimulation.^2^ This intrinsically dynamic phenomenon affects both the rate and the extent at which a system can be stimulated, further showcasing the need for time-resolved studies during dynamic stimulation.

## Conclusions

This study showcases a novel *stimulando* spectroscopic approach for investigating the effect of intermittent catalyst stimulation, with the goal of providing more efficient stimulation strategies for dynamic and resonant catalysis. In particular, we have studied the effect of intermittent UV light stimulation using vibrational spectroscopy to monitor the dynamic changes in surface chemistry during oscillation under CO_2_ hydrogenation reaction conditions for a Ni_3_–Ga/SrTiO_3_ catalyst. Rapid-scan DRIFTS allowed us to follow oscillations in surface chemistry at the stimulation limit cycle with 12.5 millisecond (80 Hz) time-resolution. The observed light-induced changes in surface chemistry under the relatively modest irradiance and photon fluxes used herein were reversible and occurred on timescales from seconds (for CO) to minutes (for formates and carbonates), with a slower change observed under dark conditions than with light. This relatively slow response in surface coverage limited the stimulation frequency at which oscillations in coverage were observed to <10 Hz, which is orders of magnitude lower than what is predicted for efficient stimulation according to resonant catalysis theory. There are several ways in which faster kinetics in the modulation of surface chemistry can be achieved, which will, in principle, depend on the catalyst, reaction, and stimulation used. It was observed that higher reaction temperatures induced faster changes in surface chemistry and increased the extent of oscillations under intermittent illumination. Higher photon fluxes may be used to increase the rate of change under irradiation, and more extreme duty cycles can give the catalyst enough time to relax during dark periods. A limitation of this study is that the catalyst material used was sluggish, and its performance did not respond to the stimulation. Other catalyst materials, such as Pt/Al_2_O_3_ and Cu–ZnO–Al_2_O_3_, showed light-induced promotion of activity, and should be the object of future studies.

## Author contributions

Floor A. Brzesowsky: conceptualization, methodology, software, investigation, data curation, formal analysis, validation, visualization, writing – original draft and writing – review & editing. Mees R. Emond: methodology, software, investigation, data curation, writing – review & editing. Jules van Leusden: methodology, writing – review & editing. Ramon Oord: methodology, writing – review & editing. Peter de Peinder: methodology, writing – review & editing. Bert M. Weckhuysen: resources, supervision, writing – review & editing. Matteo Monai: conceptualization, resources, funding acquisition, project administration, supervision, writing – original draft and writing – review & editing.

## Conflicts of interest

There are no conflicts to declare.

## Supplementary Material

FD-265-D5FD00150A-s001

## Data Availability

The data and Python scripts used for this article has been uploaded to the YODA repository and are available under: https://doi.org/10.24416/UU01-R7KP9H. Supplementary information (SI): Fig. S1–S7, including details on catalyst characterization, spectral processing, additional Diffuse Reflectance Infrared Fourier-Transform Spectroscopy (DRIFTS) analysis, infrared camera results, and gas chromatography (GC) online product analysis. See DOI: https://doi.org/10.1039/d5fd00150a.
