# Supplementary material for: Towards stimulando time-resolved infrared spectroscopy to study intermittent light-stimulated CO2 hydrogenation
Source: Faraday Discuss. 2026 Jan 6. Online ahead of print. doi: 10.1039/d5fd00150a (PMC13094466; doi:10.1039/d5fd00150a)
Supplement: FD-OLF-D5FD00150A-s001 [file FD-OLF-D5FD00150A-s001.pdf]

Supplementary Information (SI) for Faraday Discussions.
This journal is © The Royal Society of Chemistry 2026

Supporting Information to

Towards *Stimulando* Time-Resolved Infrared Spectroscopy to Study Intermittent Light-Stimulated CO_2 Hydrogenation

Floor A. Brzesowsky,[a] Mees R. Emond,[a] Jules van Leusden,[a] Ramon Oord,[a] Peter de Peinder,[a] Bert M. Weckhuysen,[a] and Matteo Monai*,[a]

[a]*Inorganic Chemistry and Catalysis group, Institute for Sustainable and Circular Chemistry, Department of Chemistry, Faculty of Science, Utrecht University, Universiteitsweg 99, 3584 CG Utrecht (The Netherlands).*

**Corresponding author: m.monai@uu.nl*

Contents

Catalyst Characterization 2
Spectral Processing 3
Additional DRIFTS analysis: Peak Area vs. Peak Intensity 3
Infrared Camera Results 4
Gas chromatography (GC) Online Product Analysis 6
Fourier Transform Analysis of IR Peak Position Shift under Intermittent UV Light Illumination 7
Bibliography 7

Catalyst Characterization

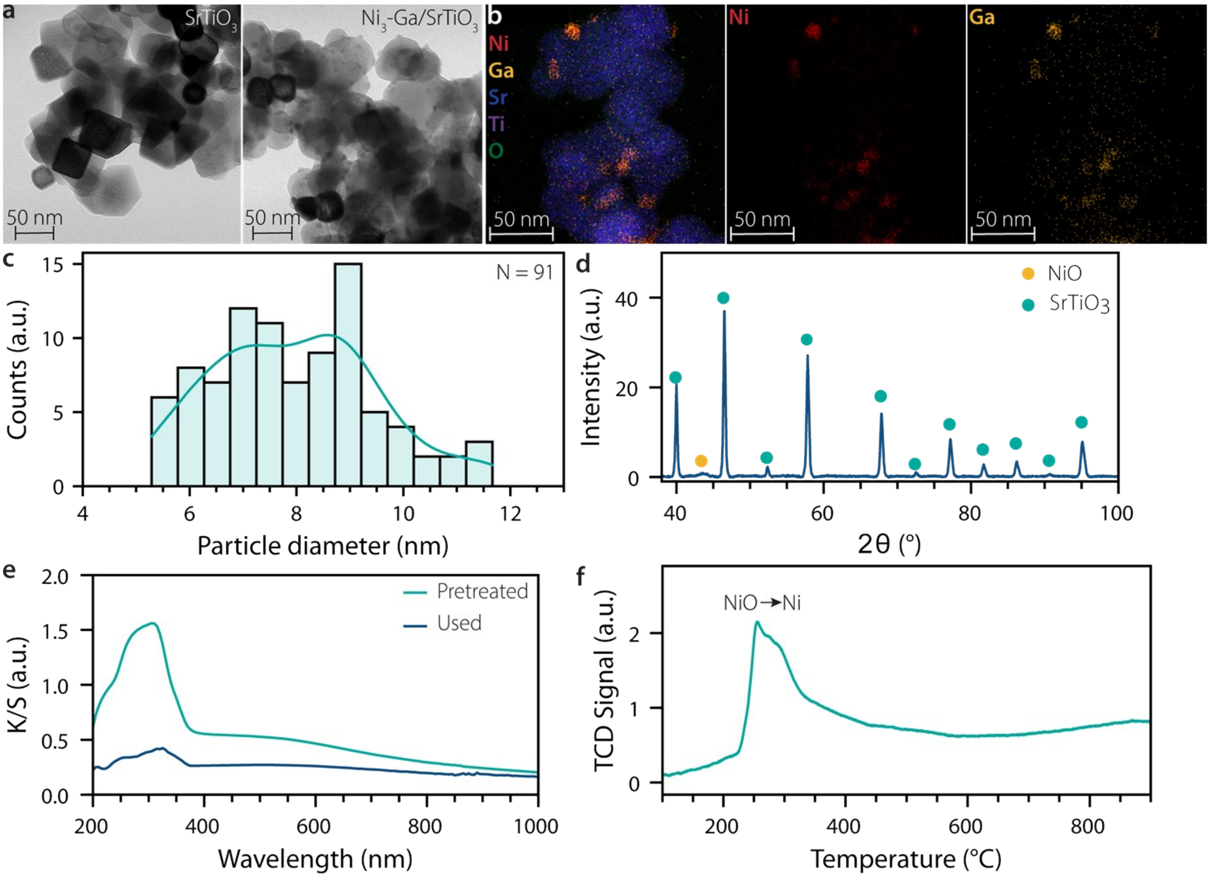

Figure S1: Catalyst characterization: A 4 wt.% Ni_3-Ga/$SrTiO_3$ catalyst was prepared by a double incipient wetness impregnation (IWI) synthesis process, based on a previously reported procedure,[1] and characterized with various analytical techniques. **a)** Transmission electron microscopy (TEM) images of the bare (left) and impregnated $SrTiO_3$ support with Ni_3-Ga (right), showing the presence of nanoparticles on the surface of the support. **b)** Energy-dispersive X-ray spectroscopy (EDX) elemental mapping of the synthesized catalyst material, showing Ni, Ga, Ti, Sr, and O maps, and Ni and Ga separately, indicating that Ni and Ga formed bimetallic particles, with some Ga segregation on the support itself. **c)** Particle size distribution of counted TEM particles (N=91) on the impregnated sample in **a** with an average of 8.0 ± 1.5 nm. **d)** X-ray diffraction (XRD) pattern of the synthesized catalyst material showing a primary contribution of the $SrTiO_3$ support and one weak reflection at 43.5° corresponding to NiO.[2] **e)** Diffuse reflectance ultraviolet-visible spectroscopy (DRS) data of the pretreated and used catalyst material (after CO_2 hydrogenation at 190 – 280 °C for 150 min.), showing ultraviolet (UV) absorbance with maximum absorbance at 305 and 320 nm, respectively. **f)** Temperature-programmed reduction (TPR) profile showing H_2 uptake below 400 °C to reduce NiO to Ni nanoparticles.

Spectral Processing

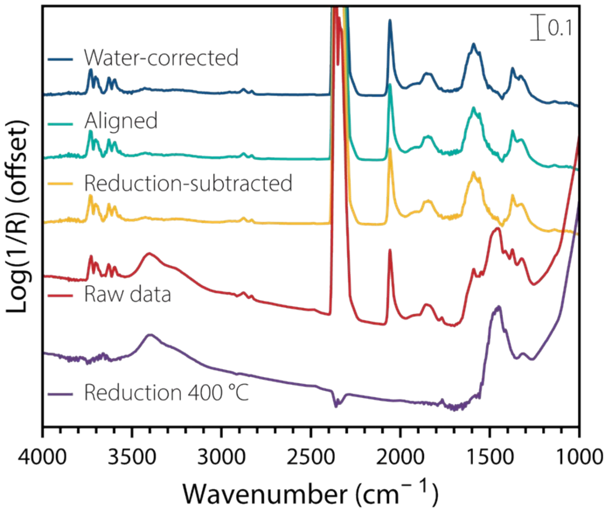

Figure S2: Spectral processing of steady-state diffuse reflectance infrared Fourier transform spectroscopy (SS-DRIFTS) and rapid scan diffuse reflectance infrared Fourier transform spectroscopy (RS-DRIFTS) data: from bottom to top: spectrum taken during reduction at 400 °C (purple), showing dominant contributions from adsorbed water, hydroxyl groups, and surface carbonates. Raw spectrum acquired during CO_2 hydrogenation at 225 °C under dark (red), showing additional gaseous CO_2, adsorbed CO, and formate bands. Subtraction of the 400 °C reduction spectrum (yellow) isolates spectral changes associated with CO_2 hydrogenation. A two-point baseline correction between 2400 – 2600 cm^{-1} (teal) was used to correct for temperature-induced baseline shifts. Final subtraction of atmospheric water rovibrational bands (dark blue) removes residual gas-phase H_2O features.[3–5]

Additional DRIFTS analysis: Peak Area vs. Peak Intensity

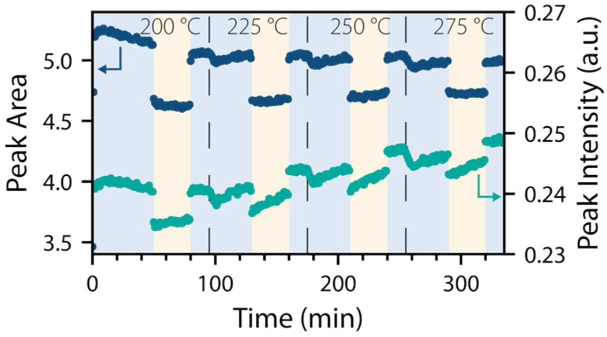

Figure S3: Comparison of Gaussian-fitted peak area (dark blue) and peak intensity (teal) over time for linearly adsorbed CO ($vCO_{lin.}$, 2055–2060 cm^{-1}) during dark and UV light periods at different reaction temperatures. Both peak area and intensity show similar behaviour under UV illumination but respond differently to temperature. Because the peak area displayed the most significant changes, it was used for further analysis. Reaction conditions: He:H_2:CO_2=20:15:5 mL/min, and p=1 atm, UV light: 385 nm LED, and 400 mW/cm^2.

Infrared Camera Results

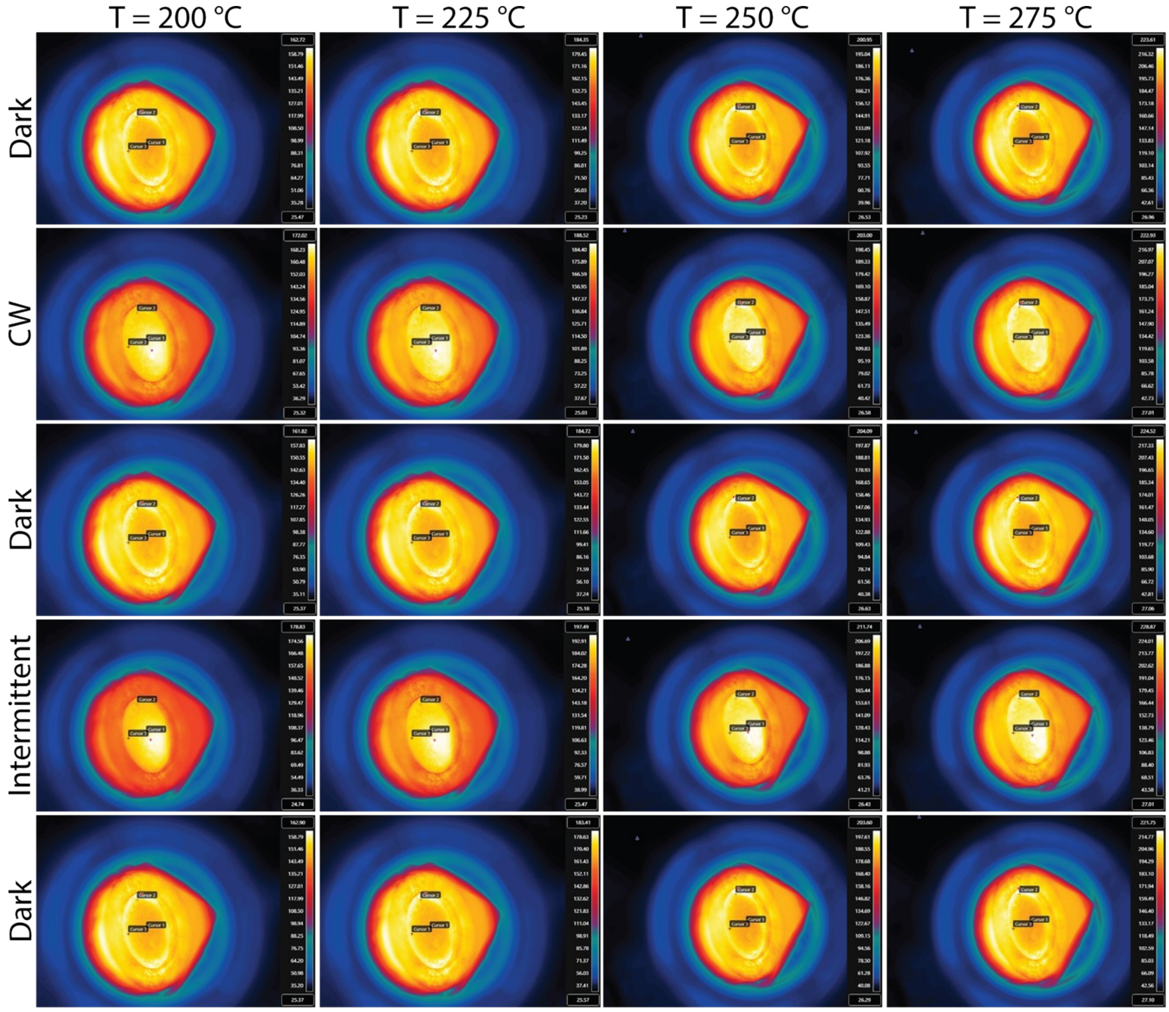

Figure S4: Infrared (IR) camera (A700 from FLIR) images recorded during light-stimulated CO_2 hydrogenation over Ni_3-Ga(4 wt.%)/$SrTiO_3$: images of the catalyst bed were collected at different reaction temperatures (i.e., 200, 225, 250, and 275 °C), with the catalyst sequentially exposed to dark, continuous (CW), dark, intermittent (1 Hz UV light) and dark again. One image was captured at each stage using an emissivity between 0.53 and 0.57, with each image displayed using a distinct colour bar. Three points are highlighted in every image, which were further analysed in **Figure S6**. The images show an increase in surface temperature of up to 40 °C during CW and intermittent UV illumination. Reaction conditions: Ar:H_2:CO_2=20:15:5 mL/min, and p=1 atm, UV light: 385 nm LED, and 400 mW/cm^2.

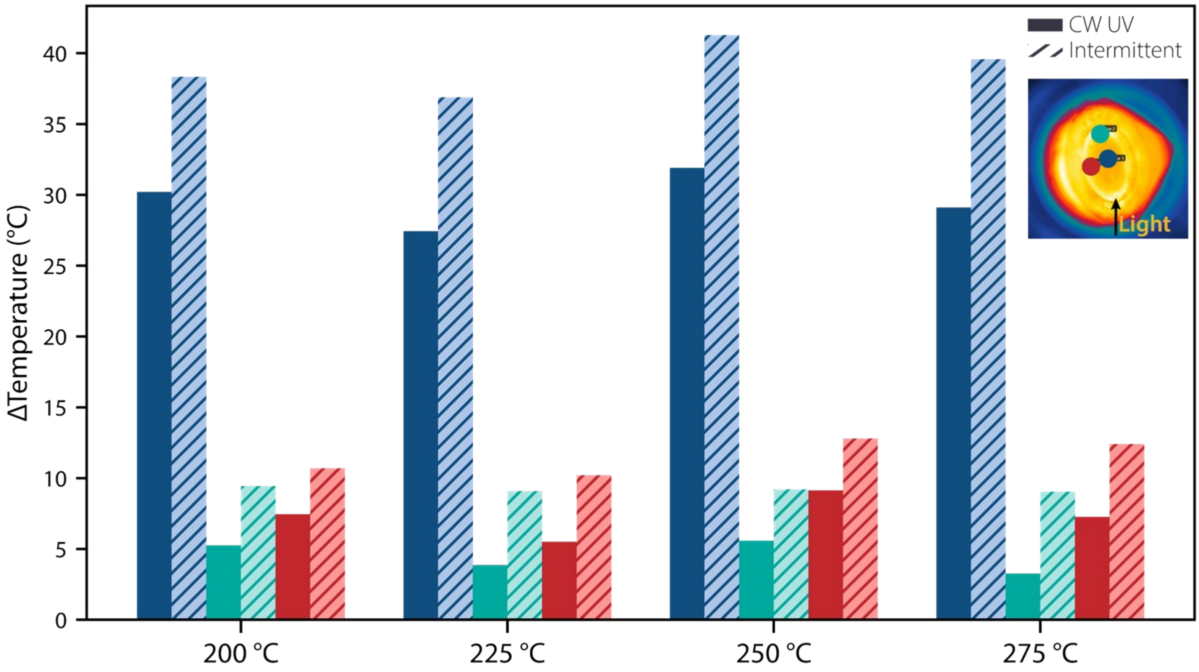

Figure S5: Analysis of the infrared (IR) camera results: Change in temperature measured at three locations on the catalyst bed: centre of the bed (dark blue), edge of the bed farthest from the light (teal), edge of the bed at the same distance from the light as the central point (red). Measurements were collected at reaction temperatures of 200, 225, 250, and 275 °C, while the catalyst was sequentially exposed to dark, continuous (CW UV), dark and intermittent (1 Hz UV) light. Temperature differences between dark and CW (solid bars), and between the second dark and intermittent illumination (dashed bars), are plotted. The central point (dark blue) exhibited the largest temperature increase, followed by the near edge (red) and the far edge (teal). Under CW UV illumination, the temperature increased by ~ 30 °C, while intermittent UV illumination resulted in an increase of ~ 40 °C. Reaction conditions: Ar:H_2:CO_2=20:15:5 mL/min, and p=1 atm, UV light: 385 nm LED, 400 mW/cm^2.

Gas chromatography (GC) Online Product Analysis

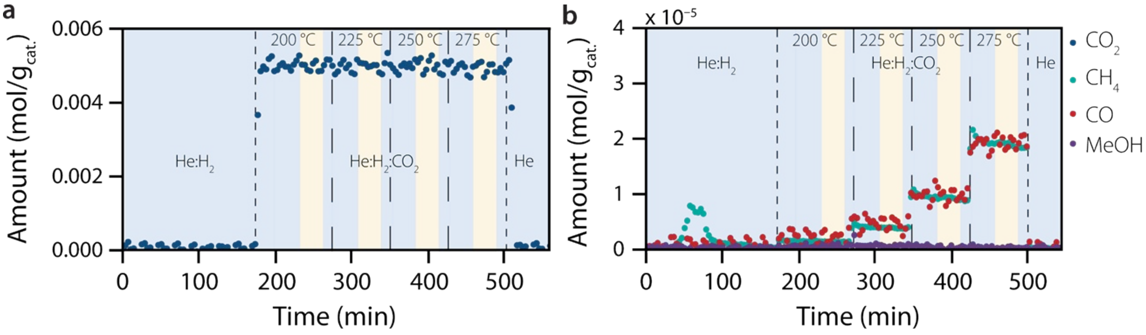

Figure S6: Online product analysis with gas chromatography (GC): a) Amount of CO_2 converted during CO_2 hydrogenation in mol per gram of catalyst material measured at different temperatures (200,225,250, and 275 °C) under dark and UV light, showing a maximum of 3 % conversion of CO_2. **(b)** Zoom-in of (a) highlighting the formation of reaction products (CH_4 and CO, with no detectable methanol). No notable changes in activity were observed under UV irradiation, except for a slight increase in methane production at higher temperatures. Reaction conditions: He:H_2:CO_2=20:15:5 mL/min, p=1 atm, UV light: 385 nm LED, and 400 mW/cm^2.

Fourier Transform Analysis of IR Peak Position Shift under Intermittent UV Light Illumination

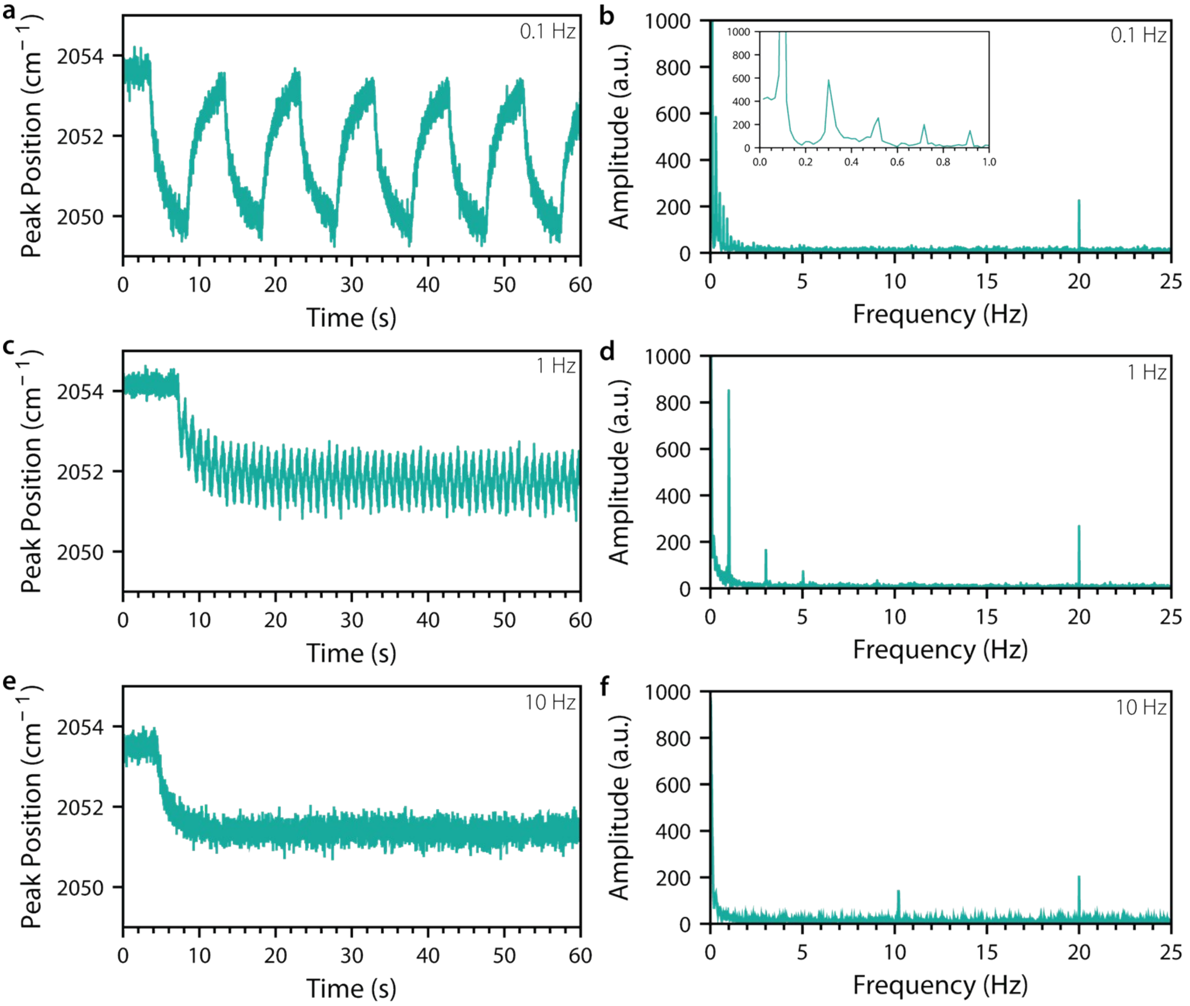

Figure S7: Frequency analysis of the IR peak position under intermittent UV illumination using different on/off frequencies. a, c, e: Change in peak position for linearly adsorbed CO ($\nu CO_{lin.}$, 2055–2060 cm^{-1}), upon switching from dark to intermittent UV light at frequencies of 0.1, 1 and 10 Hz, respectively (50 % duty cycle) at 275 °C during CO_2 hydrogenation. The peak positions were obtained from Gaussian fitting, as shown in **Figure 2**. Fourier transform analysis of these spectral changes **(b,d,f)** reveals specific frequencies at which changes are observed. Notably, overtones are observed for both 0.1 and 1 Hz stimulation, e.g. at 3 and 5 Hz in (d). All Fourier transform analyses showed a feature at 20 Hz. This frequency corresponds to the one of the interferometer mirror scanning during rapid scan diffuse reflectance infrared Fourier transform spectroscopy (RS-DRIFTS) spectra acquisition, indicating that this fast oscillation is likely an artefact from rapid scan acquisition. Reaction conditions: Ar:H_2:CO_2=20:15:5 mL/min, and p=1 atm, UV light: 385 nm LED, and 400 mW/cm^2.

Bibliography

1 F. Studt, I. Sharafutdinov, F. Abild-Pedersen, C. F. Elkjaer, J. S. Hummelshøj, S. Dahl, I. Chorkendorff and J. K. Nørskov, *Nat. Chem.*, 2014, **6**, 320–324.

2 J. F. M. Simons, Eindhoven University of Technology, 2023.

3 D. Makhmutov, E. Fedorova, A. Zanina, C. Kubis, D. Zhao, D. Doronkin, N. Rockstroh, S. Bartling, U. Armbruster, S. Wohlrab and E. V. Kondratenko, *ACS Catal.*, 2025, **15**, 2328–2341.

4 M. Kock, E. Kowalewski, D. Iltsiou, J. Mielby and S. Kegnæs, *ChemCatChem*, 2024, **16**, e202301447.

5 I. M. Hill, S. Hanspal, Z. D. Young and R. J. Davis, *J. Phys. Chem. C*, 2015, **119**, 9186–9197.
